# A Scientific Framework for Comparing Hyaluronic Acid Filler Crosslinking Technologies

**DOI:** 10.3390/gels11070487

**Published:** 2025-06-23

**Authors:** Anto Puljic, Konstantin Frank, Joel Cohen, Karine Otto, Josef Mayr, Andreas Hugh-Bloch, David Kuroki-Hasenöhrl

**Affiliations:** 1 Croma-Pharma GmbH, Cromazeile 2, 2100 Leobendorf, Austria; anto.puljic@croma.at (A.P.); karine.otto@croma.at (K.O.); josef.mayr@croma.at (J.M.); andreas.hugh-bloch@croma.at (A.H.-B.); 2 Center of Plastic, Aesthetic, Hand and Reconstructive Surgery, University Hospital Regensburg, Franz-Josef-Strauß-Allee 11, 93053 Regensburg, Germany; konstantinfrank@me.com; 3 AboutSkin Dermatology and Aesthetics, Greenwood Village, CO 80111, USA; jcohenderm@yahoo.com

**Keywords:** hyaluronic acid filler, rheology, gel content, gel particle size, extrusion force, physicochemical properties

## Abstract

Hyaluronic acid (HA) dermal fillers represent a cornerstone of modern esthetic medicine, providing a minimally invasive solution for facial volume restoration and skin rejuvenation. However, the diversity of available products, each utilizing distinct crosslinking technologies, presents a challenge for objective comparison and clinical decision making. This study introduces a scientific framework to evaluate and categorize the physicochemical properties of HA fillers based on two key parameter groups: dynamic parameters (e.g., rheology and gel content) and consistency parameters (e.g., extrusion force, water uptake, and gel particle size). Using standardized methodologies, 23 commercially available fillers from five major manufacturers were analyzed, enabling cross-technology comparisons. The findings reveal how specific crosslinking approaches influence the rheological behavior, handling characteristics, and potential clinical applications. By offering an integrated and reproducible assessment, this work helps practitioners select the most suitable filler for individualized treatment plans and encourages manufacturers to enhance product transparency through harmonized testing protocols.

## 1. Introduction

Hyaluronic acid (HA) soft tissue fillers have revolutionized minimally invasive esthetic treatments, offering a versatile and effective solution for age-related volume loss, facial contouring, and skin quality enhancement. HA is a naturally occurring glycosaminoglycan found throughout the body including in the skin, connective tissue, and synovial fluid where it plays a crucial role in maintaining hydration, elasticity, and structural integrity. Due to its high water-binding capacity and excellent biocompatibility, HA is an ideal material for injectable fillers [1,2].

HA fillers replenish endogenous HA and compensate for the loss of subcutaneous fat, restoring facial volume and improving hydration. The body’s natural HA production declines with age and thus signs such as skin laxity, wrinkles, and volume loss become more prominent in specific areas, including the nasolabial folds, marionette area, cheek, temple, and other facial areas. Injectable HA fillers effectively counteract these effects by providing immediate volume restoration and stimulating neocollagenesis, which supports long-term skin improvement [2,3,4].

Over the past decade, the landscape of HA-based soft tissue fillers has expanded significantly. In Europe alone, more than 140 HA-based facial injectables are currently approved for use [5]. In the United States, the number is comparatively lower, with more than 20 HA fillers approved by the FDA. This proliferation of products, each with unique properties and indications, presents a challenge for clinicians to stay informed and make optimal choices for patient care. Recent studies have compared various physical and chemical properties of HA fillers. Edsman et al. explored gel strength, degree of modification (MoD), and modification efficacy (MoE) [6]. Sundaram et al. conducted frequency tests at 7 Hz and 30 °C [7]. La Gatta et al. examined soluble fraction, hydrodynamic properties, rheological measurements, extrusion force, filler stability, hydration capacity, cohesivity, and stability to degradation [8,9]. Fagien et al. analyzed rheological parameters, swelling factor, and cohesion [10]. Hong et al. performed amplitude sweep tests at 37 °C [11]. Fundaro et al. assessed G′, G″, tan delta, crosslinking degree (CrD), and degradability [12]. Pluda et al. studied elasticity, dynamic viscosity, cohesivity, particle size, swelling factor, and MoD for Hyal System dermal fillers [13]. Most studies focus either on a limited set of parameters or specific products, and many are not directly comparable due to variations in testing parameters.

To enable a more systematic comparison across different crosslinking technologies, it is important to understand the common structural elements underlying HA fillers. Crosslinked HA fillers are composed of gel particles formed by HA macromolecules that are covalently bound via BDDE crosslinking. These gel particles represent the core structural units of the filler and largely determine its mechanical and handling behavior. Many of the key parameters tested in this study, such as rheological properties, gel content, swelling, and extrusion force, directly reflect the intrinsic characteristics of these gel particles. Therefore, understanding the role of gel particles is essential when comparing crosslinking technologies and their impact on product performance.

The objective of this article is to provide a structured and extensive comparison of how different crosslinking technologies influence the physicochemical properties of HA fillers. For clarity, these properties are categorized into two main groups: dynamic parameters and consistency parameters.

Dynamic parameters describe the functional behavior of a filler after injection and are tailored to meet the requirements of specific clinical indications. Key parameters include rheological properties (measured using storage modulus G′, loss modulus G″, shear stress τ) and gel content, which influence the product’s flexibility, firmness, lifting capacity, and in vivo durability [14,15]. By adjusting these characteristics, manufacturers can develop fillers suited to a broad range of esthetic applications, such as volume restoration, fine line correction, and structural support. This diversity enables practitioners to select products that align with the specific anatomical and esthetic needs of individual patients, thereby supporting predictable and effective treatment outcomes.

In contrast, consistency parameters refer to filler characteristics that directly impact the practitioner’s handling experience and are critical for ensuring reliable, repeatable application. These parameters include extrusion force, water uptake, and gel particle size, each of which affects ease of use and procedural predictability [16,17,18].

Maintaining consistent values across these factors within a specific crosslinking technology supports predictable injection performance, enabling practitioners to focus on patient specific outcomes rather than adapting to product variability. For instance, a uniform extrusion force across a product range ensures consistent handling experience, regardless of the specific filler used.

## 2. Results

The following results summarize the characterization of 23 commercially available crosslinked hyaluronic acid fillers, grouped by crosslinking technology and evaluated according to key dynamic and consistency parameters.

### 2.1. Sample Selection

All 23 HA-based soft tissue fillers, included in this study, are available in Europe, and some are also available in other countries, including the US, although some have different product names. All formulations contain lidocaine and are crosslinked using 1,4-butanediol diglycidyl ether (BDDE). The product selection comprised the leading products from five market-leading manufacturers in the esthetic injectable field: Allergan Aesthetics (AbbVie Inc., North Chicago, IL, USA), Croma (Croma-Pharma GmbH, Leobendorf, Austria), Galderma (Galderma S.A., Lausanne, Switzerland), Merz Aesthetics (Merz Pharma GmbH & Co. KGaA, Frankfurt, Germany), and Teoxane (Teoxane Laboratories, Geneva, Switzerland).

The products were categorized according to their distinct crosslinking technologies, which play a central role in defining both the manufacturing process and the physicochemical properties of the final products. Note that due to the crosslinking process, determination of the raw material molecular weight(s) in the products is not possible. An overview of the selected fillers and their classification by crosslinking technology is provided in Table 1 and Table 2.

### 2.2. Dynamic Parameter—Rheology (Storage Modulus G′)

Rheology is used to understand and predict the flow and deformation behavior of substances subjected to external forces. For dermal fillers, rheological parameters, such as the storage or elastic modulus (G′) and loss or viscous modulus (G″), and parameters such as shear stress (τ) can be evaluated using oscillatory measurements. These measurements are taken by applying either variable frequencies under a constant shear stress (or constant shear strain) or a variable shear stress (or shear strain) under a constant frequency. The first method described above is a frequency sweep and is historically the most common measurement for describing the elasticity of dermal fillers and providing curves and values such as G′. The second method, known as an amplitude sweep, allows the determination of additional information such as the structural stability or the occurrence of yielding and flowing. Both methods provide specific curves of G′ and G″. Frequency sweeps assess how these moduli change across different oscillation frequencies, thereby reflecting the material’s short-term (high-frequency) and long-term (low-frequency) deformation behavior. In contrast, amplitude sweeps evaluate how the filler structure responds to increasing strain and are used to define the linear viscoelastic region (LVR), the range in which G′ and G″ remain constant. Frequency sweep must be performed within the LVR established by the amplitude sweep to ensure valid reproducible results. In this study, both amplitude and frequency sweeps were conducted for all products. As G″ values are consistently lower than G′ in crosslinked dermal fillers and offer limited interpretative value, they were not included in this analysis (for completeness, G″ and tan δ values are reported in Table A1).

**Figure 1:**
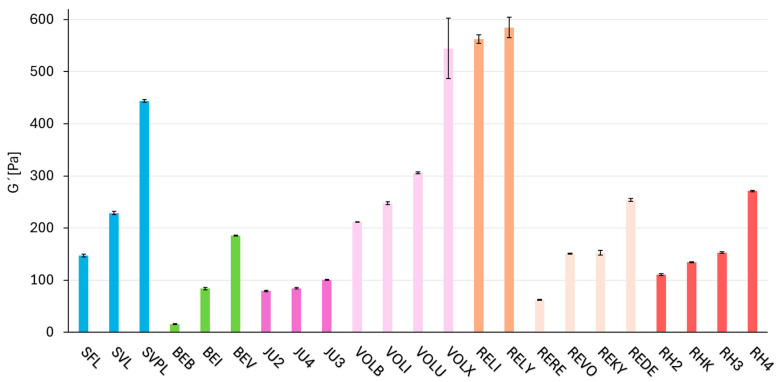
Storage modulus G′ of different dermal filler products determined at 1 rad/s. A total of 23 different dermal fillers from five different manufacturers were categorized according to their crosslinking technology and their rheological characteristics were evaluated. Products based on the same crosslinking technology are shown in the same color and arranged in order of increasing G′. The standard deviation is indicated by black error bars for each product.

The G′ values are correlated with the pressure applied to the gel and its resistance to deformation. Figure 1 and Table A1 present the G′ values for all tested products. Each product was measured in triplicate, and the standard deviations of these measurements are depicted by the error bar in the respective figure. Within each crosslinking technology (see Table 1), every single product is ordered from lowest to highest G′ values, and this ordering is consistently maintained throughout the subsequent figures and tables in the Results section. Within most technologies, there are differences in G′ values for individual products, in order to cover the various intended uses. Macro and Vycross technologies cover the widest G′ range, while the products within the NASHA and Hylacross technologies exhibit the narrowest. The complete frequency sweeps are shown in Figures S1–S7.

### 2.3. Dynamic Parameter—Rheology (Shear Stress τ)

The shear stress (τ) value at the end of the LVR (linear viscoelastic range) represents the pressure that causes the irreversible deformation of the HA filler. A product with a high τ can withstand higher deformation and still return to its original shape [24].

**Figure 2:**
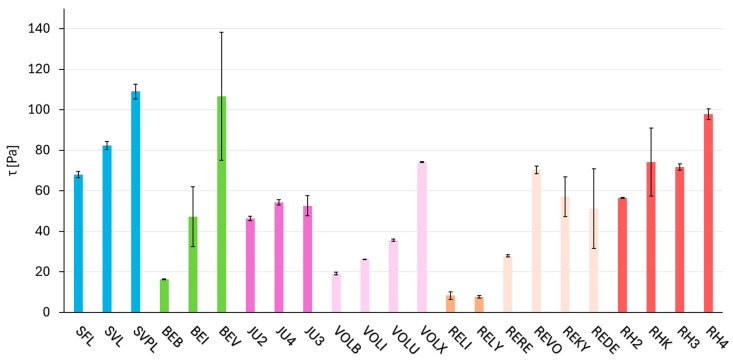
Shear stress (τ) at the end of the LVR (linear viscoelastic range) measured using the amplitude sweep test. The fillers are categorized according to their crosslinking technologies; products based on the same crosslinking technology are shown in the same color and arranged in order of increasing G′ (storage modulus). The standard deviation is indicated by black error bars for each product.

The results shown in Figure 2 and Table A2 represent τ at the end of the LVR, measured using an amplitude sweep test. Each product was measured three times, and the standard deviations of these measurements are depicted by the error bars in the respective figure. For most of the crosslinking technologies, τ increases with an increase in G′, as measured by the frequency test (see Figure 2). For Hylacross and NASHA, it was observed that both τ and G′ (see Figure 1) fall within a relatively narrow range and display only minimal deviation from each other within the respective crosslinking technologies. Macro and RHA exhibit the highest mean τ and DCLT covers the biggest range of τ. The complete amplitude sweeps are shown in Figures S8–S14.

### 2.4. Dynamic Parameter—Gel Content

**Figure 3:**
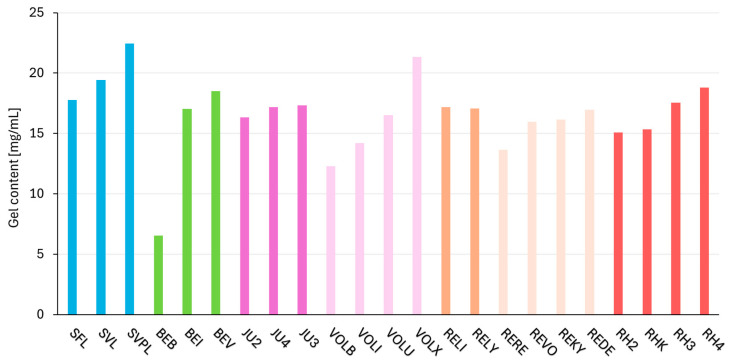
Results of the gel content determination in mg/mL. The fillers are categorized according to their crosslinking technologies; products based on the same crosslinking technology are shown in the same color and arranged in order of increasing G′ (storage modulus).

The gel content measurement results (in %) are summarized in Table A3. As the products contain different amounts of HA (see first column of Table A3; total HA content [mg/mL] as provided by the manufacturer), the relative amount of gel in the filler (in %) is not suitable for comparison. Therefore, the gel content in mg/mL is depicted, which is the result of multiplying the total HA content and the gel content in %. Only this value will be discussed further. Figure 3 illustrates this for each product and crosslinking technology. The products with the highest amounts of gel are SVPL (22.5 mg/mL), VOLX (21.3 mg/mL), and SVL (19.4 mg/mL). BEB had the lowest gel content at 6.5 mg/mL.

### 2.5. Consistency Parameter—Extrusion Force

**Figure 4:**
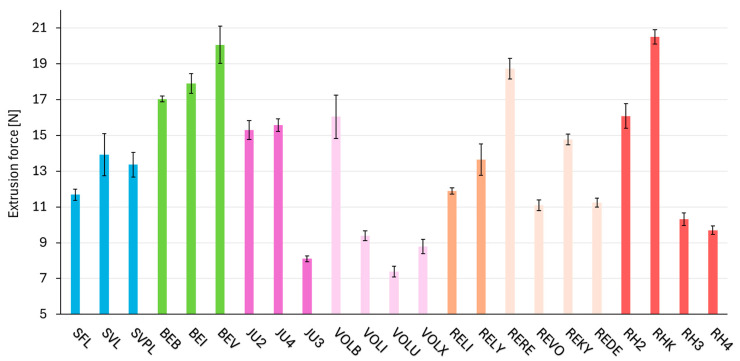
Extrusion force measured using the needle provided with each respective product. The fillers are categorized according to their crosslinking technologies; products based on the same crosslinking technology are shown in the same color and arranged in order of increasing G′ (storage modulus). The standard deviation is indicated by black error bars for each product.

The extrusion forces presented in Figure 4 and Table A4 illustrate variations among the different HA crosslinking technologies. Macro (extrusion force range of 12–14 N) and NASHA (12–14 N) exhibit the narrowest range of extrusion forces, followed by DCLT (17–20 N). In contrast, RHA (10–21 N) and Vycross (7–16 N) display the widest range of extrusion forces within a single crosslinking technology. In a comparison of JU3 and VOLU, the two products with the lowest measured extrusion forces both tested with the same needle type further illustrates that extrusion force does not directly correlate with G′. Despite their similar extrusion force, VOLU exhibits a G′ value nearly three times higher than that of JU3, indicating that matrix stiffness alone does not determine extrusion behavior.

### 2.6. Consistency Parameter—Water Uptake

**Figure 5:**
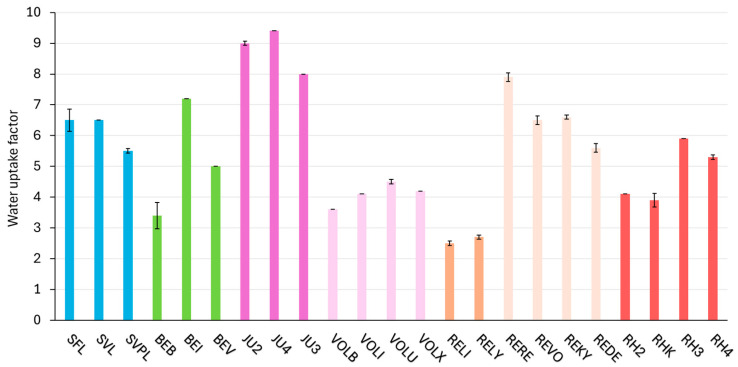
Water uptake measured for the tested HA (hyaluronic acid) fillers (mean and standard deviation). The fillers are categorized according to their crosslinking technologies; products based on the same crosslinking technology are shown in the same color and arranged in order of increasing G′ (storage modulus). The standard deviation is indicated by black error bars for each product.

Figure 5 presents the water uptake results for all tested products, highlighting differences across the products. NASHA demonstrated the lowest water uptake factor range (2.5–2.7), whereas Vycross exhibited the highest water uptake factor, reaching up to 9.4. Within NASHA, Hylacross, and Macro crosslinking technologies, the variation between the lowest and highest products was one (1) or less. In contrast, a notable difference of 3.8 was observed among the DCLT products, between BEB (lowest) and BEI (highest). It is important to note that in vitro water uptake data do not directly translate to in vivo swelling behavior, as clinical outcomes are influenced by additional factors such as tissue integration, enzymatic degradation, and individual hydration status.

### 2.7. Consistency Parameter—Gel Particle Size Distribution

**Figure 6:**
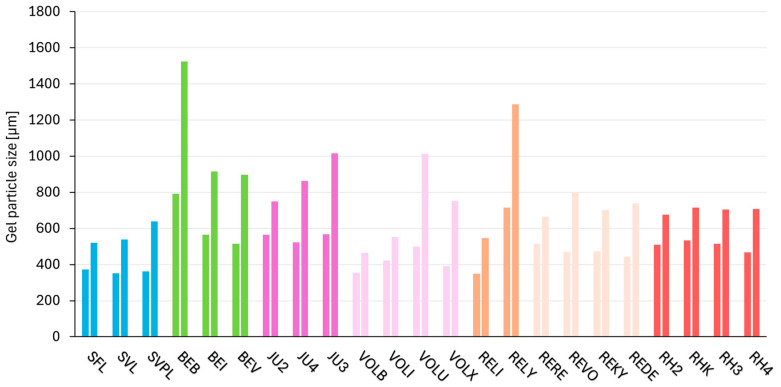
Gel particle size distribution of DV50 (median gel particle diameter (50% of particle volume is below this size) (left)) and DV90-10 (difference between DV90 and DV10 (particle size range of the central 80% of the volume) (right)). The fillers are categorized according to their crosslinking technologies; products based on the same crosslinking technology are shown in the same color and arranged in order of increasing G′ (storage modulus).

The results of the gel particle size distribution are depicted in Figure 6 and Table A5. DV50 represents the median gel particle diameter (50% of particle volume is below this size) and DV90-10 (represents the difference between DV90 and DV10 (particle size range of the central 80% of the volume)). For NASHA and DCLT, the median gel particle diameter with 50% of sample volume below the size (DV50) varied the most within each respective crosslinking technology. The Macro technology demonstrates the narrowest range (351–374 µm) for DV50.

## 3. Discussion

The available literature contains many different values, parameters, and test methods intended to describe HA-based dermal fillers. However, these methods are often designed in such a manner that they are only suitable for individual crosslinking technologies within a specific manufacturer, making it almost impossible to compare them [6,7,8,9,10,11,12,13].

The aim of this study was to conduct transparent and comparable tests across product groups using different crosslinking technologies and compare their physicochemical parameters. It is essential to acknowledge that complex products such as HA fillers cannot be adequately characterized by a single parameter, given their rheological non-Newtonian nature. Moreover, fillers consist of two different types of HA: crosslinked and non-crosslinked. It is therefore important to explain the behavior of such complex systems by comparing not only one but an entire set of parameters [12]. For instance, as explained below, some of these parameters can have direct clinical relevance. Extrusion force and flow characteristics can translate to the pressure needed to deliver the filler. Water-binding in vitro can translate to anticipated likelihood of swelling in vivo.

To further improve our understanding of the complex interrelationships between the different investigated parameters, they were consequently categorized into two groups: consistency parameters influencing the application experience for the practitioner, and dynamic parameters enabling tailor-made product features for the various use cases and applications. Similarly, parameters like extrusion force are reliable and easy to use for practitioners when kept consistent across the products, thus ensuring a predictable handling experience regardless of the product selected. In this systematic approach to product development, crosslinking technology plays a crucial role in ensuring that the final product addresses diverse clinical needs while maintaining a high level of usability and performance consistency.

Historically, the rheological parameter G′ has been used to predict the clinical efficacy and correlate with the in vivo lift capacity of the filler [14,25,26,27]. The stiffness or hardness of HA filler, described by its elasticity through the storage modulus G′, is usually correlated in the literature with the lifting capacity and volumizing effect. Thereby, the presented G′ values are mostly interpolated from frequency sweeps at low frequencies, i.e., at nearly static conditions, which is physiologically relevant according to the authors [14,28,29,30].

However, Hee and his colleagues showed that the correlation of G′ and lift capacity is only verified for in vivo values within the same crosslinking technologies. In view of this conclusion, the relevance of the G′ values needs to be evaluated carefully. Most technologies display an increasing G′ for products that need a higher lift capacity in accordance with their intended use. This correlation is valid for the Macro technology, since G′ is different for all of the products. The same conclusion regarding the correlation can be drawn for the DCLT, Vycross, OBT, and RHA technologies. While NASHA products have G′ values within a narrow range, clinical versatility within this technology is achieved by modulating gel particle size distribution, a strategy that aligns with the findings of this study [6].

Note that the distribution of G′ values within a technology group cannot be correlated with the effectiveness of the crosslinking but seems to be a strategic selection of the manufacturers according to the intended use of the proposed product range.

Moreover, G′ evaluated in static conditions does not fully represent the dynamic environment of the layer structure of the skin. On one hand, it is essential to know the properties at rest to predict the filling effect of a filler. On the other hand, movements caused by facial expressions like flexing and stretching surely have an influence on the implanted filler. A review of the extant literature reveals a considerable number of publications from various other groups that have also published similar parameters to complement static measurements. This is a further indication that other groups have also recognized the need to describe HA fillers with additional parameters [11,24,29].

Therefore, understanding the behavior of the filler when subjected to facial movement is as crucial as its characterization under static conditions. To complement the well-established value of G′ measured using a frequency sweep, we aimed to introduce an additional parameter measured using an amplitude sweep. This new parameter is intended to reflect properties like deformation under dynamic conditions, thereby providing supplementary insights into the widely recognized value of G′. Amplitude sweeps generally aim to describe the deformation behavior in the non-destructive deformation range. They were measured here using the LVR (linear viscoelastic range). The shear stress (τ), which is a key rheological parameter describing the ability of samples to withstand stress and still return to their original shape without structural change, was determined at the end of the LVR. The Macro technology, followed by RHA, exhibits the highest τ values when considering the whole crosslinking technology. Among individual products from other technologies, only BEV, VOLX, and REVO can compete with these high τ values. Although rheological measurements cannot accurately simulate the behavior of implanted dermal fillers due to the different environmental conditions in the instrument and in the skin, they can provide a reliable comparative performance The higher τ is, the more the filler can be deformed without losing its stiffness. For larger deformation the filler may be irreversibly deformed, especially after the crossover point.

The gel content specified in this study represents the quantity of gelatinous (crosslinked) HA in the tested sample. It should not be confused with the (total) HA content mentioned in the instructions for use of the tested product, which includes both crosslinked and non-crosslinked HA. Although non-crosslinked HA has beneficial properties related to skin hydration and quality, it does not contribute to the long-term lifting performance of the filler, as non-crosslinked HA is rapidly degraded within days to weeks in the skin. For fillers designed to provide a durable lifting effect, a high gel content is desirable [31]. As shown in Figure 6, most of the products have comparable high absolute amounts of gel (17 of 23 products within 15–19 mg/mL). Nonetheless, the small differences within the products of a crosslinking technology still correlate with G′ values (see Vycross, Macro, OBT, DCLT, and RHA). In the case of Hylacross and NASHA products, the G′ values within a crosslinking technology were not differentiable; also, the amount of gel was uniform. Additionally, Table A3 shows that the HA content of the fillers is not directly correlated with the amount of crosslinked HA within the fillers of a crosslinking technology. Some products have uniform gel content (in %) and only have increasing amounts of total HA (Vycross); others keep the total HA concentration constant and have increasing gel content (OBT and RHA) and some fillers have both increased (Macro and DCLT). Solely, Hylacross and NASHA products have uniform HA and gel content within the product line. In summary, all of the crosslinked, dermal fillers have a high amount of gelatinous, crosslinked HA (>65%; except for BEB), which is in accordance with the general knowledge about high amounts of crosslinked HA being advantageous for high lifting capacities and prolonged persistence [32,33,34,35].

The force required to extrude the product from the syringe with the provided or recommended needle is one of the properties that practitioners experience most directly, and it is therefore important and necessary to be as comparable and, above all, predictable as possible within a crosslinking technology. A consistent extrusion force across a crosslinking technology enables practitioners to develop a more intuitive injection technique, ultimately enhancing procedural efficiency and patient comfort. Variability in extrusion force between different fillers within a crosslinking technology can lead to unexpected challenges during application, potentially impacting the precision of placement and increasing the risk of inconsistent outcomes. The products that were analyzed (Figure 4) in the study reveal a substantial variation in the force required for application, ranging from 7 N to 20 N. This finding highlights the necessity for users to be able to accurately determine the required force for each product prior to its injection into the patient. As demonstrated in Figure 4, the crosslinking technologies Macro, DCLT, and NASHA exhibited minimum deviations for their products, with all three showing a maximum spread in extrusion force of no more than 3 N.

The clinical significance of extrusion force for esthetic practitioners cannot be overstated. A higher extrusion force can make the injection process more challenging, especially during delicate procedures requiring fine control, such as superficial injections or treatments in areas with thin skin. Increased force can also lead to a sudden release or “bursting” of the filler when the pressure threshold is exceeded, potentially causing irregular placement or overcorrection. Conversely, a very low extrusion force might compromise precision by reducing tactile feedback during injection. Consistency in extrusion force within a crosslinking technology therefore allows practitioners to anticipate the handling properties of different products more reliably, leading to smoother injections, improved ergonomic comfort, and ultimately better esthetic outcomes for patients. From a training perspective, predictable extrusion behavior also aids in shortening the learning curve for new practitioners adopting a particular technology. Therefore, minimizing intra-technology variations in extrusion force directly contributes to practitioner confidence, procedural safety, and patient satisfaction.

The water uptake ratio, measured after 72 h, is another key parameter impacting user reliability. This measurement quantifies the filler’s maximum water uptake, providing information which may not be immediately apparent to practitioners who typically only observe instant swelling occurring within a few hours post-injection [16]. This parameter is particularly important for practitioners, as they must adjust the amount of fillers injected based on the expected volume increase. Consequently, a uniform water uptake ratio within a crosslinking technology offers a significant advantage, allowing it to better predict the water uptake of different products. However, it is important to note that in vitro water uptake data do not directly translate to in vivo behavior. Nonetheless, most injectors believe that some degree of correlation exists, and products that are closer to equilibrium hydration tend to exhibit less swelling. The complex biological environment, including factors like tissue interaction, enzymatic activity, and individual patient variability, can influence the actual hydration and resulting volume of the filler after injection. When comparing water uptake with other parameters, no strong or consistent correlation was observed across the tested fillers. However, the crosslinking technology with the highest G′ values, NASHA (562–585 Pa), also exhibited the lowest water uptake (2.5–2.7). In contrast, Hylacross products showed relatively low G′ values (79–100 Pa) and the highest water uptake (8.0–9.4). This observation is consistent with previous findings by Fagien et al., which also reported an inverse relationship between G′ and water uptake [10]. Notably, this correlation was only apparent at the extremes and did not hold consistently across product ranges with smaller differences. Overall, these findings suggest that water uptake is primarily influenced by the specific crosslinking architecture rather than by any single physicochemical parameter.

A further consistency parameter is gel particle size distribution. Crosslinked HA soft tissue fillers consist of a conglomerate of HA particles, which are macromolecules of BDDE-crosslinked HA. These gel particles are rigid, tough, and elastic, and the overall performance of the filler is primarily determined by their intrinsic properties. Individual particles are held together by weaker, non-covalent forces, influencing the structural integrity of the filler. When designing a crosslinking technology for HA fillers with respect to gel particle size, two primary approaches can be adopted. The first involves varying the gel particle size between individual fillers, using smaller gel particles for softer and larger gel particles for stronger fillers. The second approach is to maintain a consistent gel particle size while modifying the intrinsic properties of the gel particles to differentiate products. As shown in Figure 6, most technologies work with the second approach, i.e., keeping the gel particle size uniform. Only the DCLT and NASHA technology have more pronounced changes in particle size. RHA and Macro technology in particular manage to achieve a narrow gel particle size distribution for their crosslinking technology, as shown with the DV90-10 values in Table A5. This indicates a well-defined particle size and reflects a highly controlled and precise manufacturing process.

## 4. Conclusions

This in-depth analysis of current filler properties provides a comprehensive and comparative assessment of HA dermal fillers across a wide spectrum of crosslinking technologies. By introducing a structured framework that distinguishes dynamic from consistency parameters, we clarify how formulation choices impact physical behavior. The results demonstrate that the rheological characteristics, gel content, extrusion force, water uptake, and particle size distribution vary not only between manufacturers but also within product families, reflecting the diversity of intended clinical applications. Importantly, the categorization into dynamic (performance-driven) and consistency (handling-driven) attributes offers practical value for esthetic practitioners, enabling more informed product selection and enhanced treatment precision. This framework also sets a foundation for standardized testing and transparent communication in product development.

## 5. Materials and Methods

### 5.1. Sample Selection and Data Analysis

Products tested in this study are shown in Table 1 and were selected based on the criteria described under Section 2.1.

Unless otherwise specified, all data are means ± standard deviation. The quantity of the sample analyzed was dependent on the methodology employed. The evaluation of each crosslinking technology included a statistical comparison of the products with the lowest and highest G′ and τ values. This analysis utilized a two-sample *t*-test at a 95% confidence level under the assumption of equal variances, using Minitab version 21.1.1. The NASHA crosslinking technology was the only one that did not show statistically significant differences for either G′ or τ.

### 5.2. Rheology

The frequency sweep was performed at a constant shear strain (also called shear deformation) of γ = 1% over the angular frequency range ω of 628 to 0.2 rad/s. G′ and G″ were interpolated under nearly static conditions at an angular frequency of ω = 1 rad/s (0.16 Hz) at 25 °C, which is similar to values used in previous studies and within the LVR for all measured products [7,14,27,30]. The amplitude sweep was conducted at a constant angular frequency of ω = 10 rad/s (1.6 Hz) at 25 °C over the shear strain range γ of 0.1 to 1000%.

Both tests were performed using an Anton Paar (Graz, Austria) MCR 102 rheometer with a cone plate setup (1° cone angle, 50 mm diameter, 0.1 mm gap). Each test was performed in triplicate, using fresh samples for each measurement. The end of the LVR was defined as a 5% decrease in the G′ curve from its plateau value. The values of G′ and G″ and the values of the shear strain (γ) and shear stress (τ) at the end of the LVR were evaluated for all samples. Since shear strain (γ), commonly used in oscillatory rheological measurements, is difficult to directly correlate with real-life tissue movements, an alternative parameter was considered more suitable for describing the shear force experienced by the measured sample. This parameter is the shear stress (τ), which is directly related to the applied shear force and can be calculated from G′, G″, and γ.

(1) $$\tau =\sqrt{G^{\prime 2}+G^{\prime \prime 2}}*\gamma$$

τ represents the resistance of the sample to deformation under applied force, whereas γ reflects the relative displacement of the upper measurement plate in the rheometer.

### 5.3. Gel Content

In this study, gel content is defined as the proportion of gelatinous (crosslinked) HA, which is retained by a 0.2 µm filter, expressed as a percentage of total HA content [6]. It is determined by quantifying the concentration of the extractable, free (non-crosslinked) HA in the product and calculation of the gelatinous fraction by subtraction from the total HA content. Subsequent division by the total HA content and multiplication by 100 yields the relative gel content in percent (see Equation (2); the results are in Table A3).

Samples were prepared and analyzed in triplicate. A defined sample quantity, normalized to the HA content of the respective crosslinked product, was transferred to a volumetric flask which was then filled with a 0.9% NaCl solution and homogenized using a magnetic stirrer. The resulting suspension was then transferred to a centrifuge vial and left to stand to allow sedimentation. After sedimentation, an aliquot of the supernatant was filtered through a 0.2 µm filter and the HA content of the filtrate, which contains the extractable, non-crosslinked HA, was determined via carbazole assay, based on the method described in Ph. Eur. 01/2023:1472. The relative standard deviation (RSD%) for triplicate measurements was evaluated and found to be below 10% (≤4.1%), showing a good analytical repeatability.

(2) $$\frac{HA\;Content\left(total\right)\left[\frac{mg}{mL}\right]-HA\;Content\left(\mathrm{non-crosslinked}\right)\left[\frac{mg}{mL}\right]}{HA\;Content\left(total\right)[\frac{mg}{mL}]}\times 100=Gel\;Content\;[\%]$$

### 5.4. Extrusion Force

The extrusion force was measured using a Mecmesin (Slinfold, West Sussex, UK) Advanced Force Gauge device AFG 250 N, mounted on a test stand. This device determines the average force required to expel an HA filler from a syringe at a constant speed of 12 mm/min. The initial approximately 10 s of each measurement, corresponding to the break-loose force, were excluded from the mean force calculation, as this region does not reflect the steady-state extrusion behavior. For each product, at least three independent tests were performed, each using a new syringe and the respective needle provided in the product’s procedure pack. If a product was provided with multiple needle types, the needle with the higher nominal gauge was used for testing.

### 5.5. Water Uptake

The water uptake method was performed based on the method described by Edsman et al., with each product being measured twice [6]. This method was chosen as it is advantageous to gravimetric methods when measuring gels with a non-crosslinked HA content, which all the tested products have. A sample of 0.4–0.6 g of product was transferred through a 27 G needle (27 G ½″ TW Terumo Europe (N.V. Leuven, Belgium) K-Pack II) into a graduated 10 mL sample tube (Brand 10 mL NS 12/21; graduations: 0.1 mL). Afterwards, 7 mL of phosphate-buffered saline (PBS) was added and the sample was vortexed until the gel was fully dispersed. To ensure the complete inclusion of gel particles adhering to the tube wall, an additional 7 mL of PBS solution was added. The samples were inspected after 72 h, and the volume of the swollen gel was determined visually using the graduation of the sample tube. All experiments were conducted at ambient temperature, and a density of 1 g/cm^3^ was assumed for all products. The water uptake factor was calculated as *V*/*V*_0_, where *V* is the volume of the fully swollen gel and *V*_0_ is the volume of the product prior to PBS addition.

### 5.6. Gel Particle Size Distribution

HA fillers consist of crosslinked HA particles suspended in a matrix of non-crosslinked amorphous HA. The particle size distribution of the crosslinked fraction is an important parameter for characterizing the final product. Gel particle size distribution was determined using a laser diffraction particle size analyzer (Malvern (Worcestershire, UK) Mastersizer 3000) equipped with a Hydro MW dispersion unit. The samples were extruded using the respective needle supplied with the product (see Table A6 (for BEB and BEV, two different needles were supplied with the product and for both samples the 27 G needles were used)) and dispersed in phosphate-buffered saline (PBS) for analysis. The parameters DV10, DV50, and DV90 were determined, where DV represents the cumulative volume distribution. The gel particle size distribution is volume-weighted and reflects the size of crosslinked gel particles. DV10, DV50, and DV90 represent the particle diameters below which 10%, 50%, and 90% of the total sample volume is contained, respectively. The difference between DV90 and DV10 (DV90-10) represents the particle size range encompassing the central 80% of the sample volume.

## Figures and Tables

**Table 1:** Studied HA fillers classified according to their manufacturers and their respective crosslinking technology.

Manufacturer	Crosslinking Technology	Product Name	Abbreviation
Allergan Aesthetics	Hylacross	Juvéderm Ultra 2	JU2
		Juvéderm Ultra 3	JU3
		Juvéderm Ultra 4	JU4
	Vycross	Juvéderm Volbella	VOLB
		Juvéderm Volift	VOLI
		Juvéderm Voluma	VOLU
		Juvéderm Volux	VOLX
Croma	Macro	Saypha Filler Lidocaine	SFL
		Saypha Volume Lidocaine	SVL
		Saypha Volume Plus Lidocaine	SVPL
Galderma	NASHA	Restylane Lidocaine	RELI
		Restylane Lyft Lidocaine	RELY
	OBT	Restylane Refyne	RERE
		Restylane Kysse	REKY
		Restylane Volyme	REVO
		Restylane Defyne	REDE
Merz Aesthetics	DCLT	Belotero Balance Lidocaine	BEB
		Belotero Intense Lidocaine	BEI
		Belotero Volume Lidocaine	BEV
Teoxane	RHA	Teosyal RHA 2	RH2
		Teosyal RHA 3	RH3
		Teosyal RHA 4	RH4
		Teosyal RHA Kiss	RHK

**Table 2:** Description of studied HA fillers’ respective crosslinking technology.

Crosslinking Technology	Crosslinking Description
Hylacross	HAs of the same molecular weight are crosslinked together, creating a relatively uniform product of set viscosity and thickness [19].
Vycross	Utilizes a novel mix of low-molecular-weight HA (<1 MDa) and high-molecular-weight HA (>1 MDa); low HA concentration [20].
Macro	Predefined viscoelastic characteristics. High molecular weight HA undergoes controlled processing to maintain consistency in the crosslinking process [21]
NASHA	Preservation of naturally long HA chains resulting in strong gels with high G′. Minimal modification and controlled particle sizing [22].
OBT	Flexible HA Fillers. Strength/firmness varied by applying different degrees of crosslinking [22].
DCLT	DCLT includes two additional phases. This provides a combination of cohesivity, elasticity, and plasticity [23].
RHA	Improved HA chain integrity, preserving long (high Mw) HA, with low amounts of crosslinker to achieve clinically desirable mechanical properties and durability [24].

## Data Availability

The original contributions presented in this study are included in the article/Supplementary Material. Further inquiries can be directed to the corresponding author.

## Supplementary Materials

The following supporting information can be downloaded at https://www.mdpi.com/article/10.3390/gels11070487/s1. Figure S1: Frequency sweep of the Hylacross products; Figure S2: Frequency sweep of the Vycross products; Figure S3: Frequency sweep of the NASHA products; Figure S4: Frequency sweep of the OBT products; Figure S5: Frequency sweep of the RHA products; Figure S6: Frequency sweep of the DCLT products; Figure S7: Frequency sweep of the MACRO products; Figure S8: Amplitude sweep of the Hylacross products; Figure S9: Amplitude sweep of the Vycross products; Figure S10: Amplitude sweep of the NASHA products; Figure S11: Amplitude sweep of the OBT products; Figure S12: Amplitude sweep of the RHA products; Figure S13: Amplitude sweep of the DCLT products; Figure S14: Amplitude sweep of the MACRO products.

## Abbreviations

The following abbreviations are used in this manuscript:

HA	Hyaluronic acid
G′	Storage modulus (elastic modulus)
G″	Loss modulus (viscous modulus)
tan δ	Loss factor
τ (tau)	Shear stress
γ (gamma)	Shear deformation
ω (omega)	Angular frequency
LVR	linear viscoelastic range
DV10	Gel particle diameter below which 10% of the total sample volume is contained
DV50	Median gel particle diameter (50% of particle volume is below this size)
DV90	Gel particle diameters below which 90% of the total sample volume is contained
DV90–10	Difference between DV90 and DV10 (particle size range of the central 80% of the volume)
PBS	Phosphate-buffered saline
BDDE	1,4-Butanediol diglycidyl ether
RSD	Relative standard deviation
G	Gauge (used for needle size, e.g., 27 G)
TW	Thin wall (needle type)
UTW	Ultra-thin wall (needle type)

## Appendix A

Table A1 presents the results of the frequency sweep measurement at an angular frequency of 1 rad/s.

**Table A1:** Results of frequency sweep at angular frequency 1 rad/s.

Product	G′ [Pa]	G″ [Pa]	tan δ [-]
SFL	147.3	26.3	0.18
SVL	228.8	26.1	0.11
SVPL	443.9	45.0	0.10
BEB	15.7	12.9	0.82
BEI	83.9	33.0	0.39
BEV	185.4	44.9	0.24
JU2	79.2	21.5	0.27
JU4	84.4	17.7	0.21
JU3	100.4	21.3	0.21
VOLB	212.1	30.4	0.14
VOLI	247.4	31.6	0.13
VOLU	306.3	30.1	0.10
VOLX	544.7	58.8	0.11
RELI	562.3	123.3	0.22
RELY	584.8	107.6	0.18
RERE	62.0	14.7	0.24
REVO	150.8	16.6	0.11
REKY	152.7	17.3	0.11
REDE	253.6	25.2	0.10
RH2	110.6	34.6	0.31
RHK	134.4	38.6	0.29
RH3	153.0	29.1	0.19
RH4	270.9	40.2	0.15

Table A2 presents the results of the amplitude sweep measurement at the end of the LVR.

**Table A2:** Results of amplitude sweep at the end of linear viscoelastic range (=LVR).

Product	LVR	LVR	LVR	LVR	LVR
	γ [%]	τ [Pa]	G′ [Pa]	G″ [Pa]	tan δ [-]
SFL	36.6	68.0	176.1	46.0	0.26
SVL	32.3	82.3	245.2	47.1	0.19
SVPL	23.0	109.0	459.9	89.5	0.19
BEB	30.7	16.3	39.0	35.2	0.88
BEI	31.8	47.3	133.3	61.6	0.45
BEV	43.7	106.7	231.7	66.4	0.28
JU2	38.9	46.3	108.7	43.7	0.39
JU4	48.0	52.6	126.9	40.8	0.32
JU3	47.5	54.3	107.2	37.6	0.35
VOLB	8.3	19.2	224.0	47.4	0.21
VOLI	9.9	26.2	254.8	52.0	0.20
VOLU	11.2	35.7	309.5	43.9	0.14
VOLX	13.0	74.2	561.6	98.1	0.17
RELI	1.1	8.3	722.7	148.2	0.20
RELY	1.1	7.8	701.6	124.2	0.17
RERE	32.8	27.9	79.9	29.6	0.37
REVO	33.9	57.1	166.8	36.8	0.22
REKY	43.7	70.4	158.5	23.1	0.14
REDE	19.8	51.2	261.2	45.7	0.18
RH2	33.8	56.6	153.9	55.3	0.35
RHK	31.2	97.8	300.3	47.9	0.15
RH3	37.7	74.1	184.5	59.2	0.31
RH4	37.3	71.7	182.1	46.5	0.25

Additional data used to calculate the gel content are depicted in Table A3.

**Table A3:** HA content as stated by the manufacturer, and measured gel content expressed as a percentage of total HA and mg/mL.

Product	HA Content [mg/mL] ^1^	Gel Content [%] ^2^	Gel Content [mg/mL] ^3^
SFL	23.0	76.0	17.5
SVL	23.0	86.0	19.8
SVPL	25.0	90.0	22.5
BEB	22.5	29.0	6.5
BEI	25.5	66.8	17.0
BEV	26.0	71.3	18.5
JU2	24.0	68.1	16.3
JU4	24.0	71.5	17.2
JU3	24.0	72.2	17.3
VOLB	15.0	82.0	12.3
VOLI	17.5	81.1	14.2
VOLU	20.0	82.6	16.5
VOLX	25.0	85.3	21.3
RELI	20.0	86.0	17.2
RELY	20.0	85.4	17.1
RERE	20.0	68.3	13.7
REVO	20.0	79.8	16.0
REKY	20.0	80.7	16.1
REDE	20.0	84.9	17.0
RH2	23.0	65.5	15.1
RHK	23.0	66.8	15.4
RH3	23.0	76.2	17.5
RH4	23.0	81.7	18.8

^1^ According to the product information provided by the manufacturer. ^2^ Represents the outcome of the measurement. ^3^ Result of the multiplication of ^1^ and ^2^.

Table A4 presents the extrusion force measurement results along with the corresponding needle types used during testing.

**Table A4:** Summary of extrusion force results and corresponding needle types.

Product	Extrusion Force [N]	Needle Type Used
SFL	12	27 G ½″ TW Terumo K-Pack II
SVL	14	27 G ½″ TW Terumo K-Pack II
SVPL	13	27 G ½″ TW Terumo K-Pack II
BEB	17 ^1^	30 G ½″ TSK PRC-30013I
BEI	18 ^1^	27 G ½″ TSK HPC-27013I
BEV	20	30 G ½″ TSK PRC-30013I
JU2	15	30 G ½″ TSK HPC-30013A
JU4	16	27 G ½″ TSK HPC-27013A
JU3	8	27 G ½″ TSK HPC-27013A
VOLB	16	32 G ½″ TSK HPC-32013A
VOLI	9	30 G ½″ TSK HPC-30013A
VOLU	7	27 G ½″ TSK HPC-27013A
VOLX	9	27 G ½″ TSK HPC-27013A
RELI	12 ^1^	29 G ½″ TW Terumo K-Pack II
RELY	14	27 G ½″ TW Terumo K-Pack II
RERE	19 ^1^	30 G ½″ TSK HPC-30013I
REVO	11 ^1^	27 G ½″ TSK HPC-27013I
REKY	15 ^1^	30 G ½″ UTW Terumo K-Pack II
REDE	11 ^1^	27 G ½″ TSK HPC-27013I
RH2	16	30 G ½″ TSK HPC-30013I
RHK	21	30 G ½″ TW Terumo K-Pack II
RH3	10	27 G ½″ TSK HPC-27013I
RH4	10	27 G ½″ TSK HPC-27013I

^1^ Mean value of three measurements. All other values represent the mean of six measurements.

Gel particle size distribution data are shown in Table A5.

**Table A5:** DV10, DV50, and DV90 values from gel particle size distribution analysis.

Product	Gel Particle Size Distribution [µm]			
	DV10	DV50	DV90	DV90-10
SFL	182	374	703	521
SVL	164	351	704	540
SVPL	152	361	791	639
BEB	346	792	1870	1524
BEI	275	566	1190	915
BEV	214	514	1110	896
JU2	280	566	1030	750
JU4	236	523	1100	864
JU3	253	569	1270	1017
VOLB	174	354	639	465
VOLI	198	424	751	553
VOLU	176	498	1190	1014
VOLX	140	390	891	751
RELI	123	348	669	546
RELY	152	716	1440	1288
RERE	247	515	912	665
REVO	198	470	999	801
REKY	203	474	904	701
REDE	177	443	917	740
RH2	245	511	920	675
RHK	252	534	968	716
RH3	238	514	943	705
RH4	195	468	902	707

The HA fillers included in this study are summarized in Table A6, classified by manufacturer and crosslinking technology.

**Table A6:** Overview of HA fillers by manufacturer and crosslinking technology.

Manufacturer ^1^	Crosslinking Technology	Product Name	Abbreviation	Provided Needle	Product Batch Reference
Croma	Macro	Saypha Filler Lidocaine	SFL	27 G ½″ TW Terumo K-Pack II	905015
		Saypha Volume Lidocaine	SVL	27 G ½″ TW Terumo K-Pack II	106024
		Saypha Volume Plus Lidocaine	SVPL	27 G ½″ TW Terumo K-Pack II	206020
Merz Aesthetics	DCLT	Belotero Balance Lidocaine	BEB	27 G ½″ TSK PRC-27013I 30 G ½″ TSK PRC-30013I	B00021100
		Belotero Intense Lidocaine	BEI	27 G ½″ TSK HPC-27013I	B00030380
		Belotero Volume Lidocaine	BEV	27 G ½″ TSK PRC-27013I 30 G ½″ TSK PRC-30013I	B00021920
Allergan Aesthetics	Hylacross	Juvéderm Ultra 2	JU2	30 G ½″ TSK HPC-30013A	1000610608
		Juvéderm Ultra 3	JU3	27 G ½″ TSK HPC-27013A	1000648446
		Juvéderm Ultra 4	JU4	27 G ½″ TSK HPC-27013A	1000749866
	Vycross	Juvéderm Volbella	VOLB	32 G ½″ TSK HPC-32013A	1000594126
		Juvéderm Volift	VOLI	30 G ½″ TSK HPC-30013A	1000568917
		Juvéderm Voluma	VOLU	27 G ½″ TSK HPC-27013A	1000609303
		Juvéderm Volux	VOLX	27 G ½″ TSK HPC-27013A	1000546997
Galderma	NASHA	Restylane Lidocaine	RELI	29 G ½″ TW Terumo K-Pack II	21568-1
		Restylane Lyft Lidocaine	RELY	27 G ½″ TW Terumo K-Pack II	21775-1
	OBT	Restylane Refyne	RERE	30 G ½″ TSK HPC-30013I	21497-1
		Restylane Kysse	REKY	30 G ½″ UTW Terumo K-Pack II	21642
		Restylane Volyme	REVO	27 G ½″ TSK HPC-27013I	21641
		Restylane Defyne	REDE	27 G ½″ TSK HPC-27013I	21545
Teoxane	RHA	Teosyal RHA 2	RH2	30 G ½″ TSK HPC-30013I	23171DLO
		Teosyal RHA 3	RH3	27 G ½″ TSK HPC-27013I	23171JLO
		Teosyal RHA 4	RH4	27 G ½″ TSK HPC-27013I	23341FLO
		Teosyal RHA Kiss	RHK	30 G ½″ Thin wall Terumo K-Pack II	23221ALO

^1^ Croma (Croma-Pharma GmbH, Leobendorf, Austria), Merz Aesthetics (Merz Pharma GmbH & Co. KGaA, Frankfurt, Germany), Allergan Aesthetics (AbbVie Inc., North Chicago, IL, USA), Galderma (Galderma S.A., Lausanne, Switzerland), and Teoxane (Teoxane Laboratories, Geneva, Switzerland).
