# Supplementary material for: A Scientific Framework for Comparing Hyaluronic Acid Filler Crosslinking Technologies
Source: Gels. 2025 Jun 23;11(7):487. doi: 10.3390/gels11070487 (PMC12294425; doi:10.3390/gels11070487)
Supplement: Supplementary file 1 [file gels-11-00487-s001.zip › gels-3684404-supplementary.pdf]

Supplementary Materials

# A Scientific Framework for Comparing Hyaluronic Acid Filler Crosslinking Technologies

**Anto Puljic [1], Konstantin Frank [2], Joel Cohen [3], Karine Otto [1], Josef Mayr [1], Andreas Hugh-Bloch [1] and David Kuroki-Hasenöhrl [1,*]**

[1] Croma-Pharma GmbH, Cromazeile 2, 2100 Leobendorf, Austria; anto.puljic@croma.at (A.P.); karine.otto@croma.at (K.O.); josef.mayr@croma.at (J.M.); andreas.hugh-bloch@croma.at (A.H.-B.)
[2] Center of Plastic, Aesthetic, Hand and Reconstructive Surgery, University Hospital Regensburg, Franz-Josef-Strauß-Allee 11, 93053 Regensburg, Germany; konstantinfrank@me.com
[3] AboutSkin Dermatology and Aesthetics, Greenwood Village, CO 80111, USA; jcohenderm@yahoo.com
* Correspondence: david.hasenoehrl@croma.at; Tel.: +43-676-846-868-161

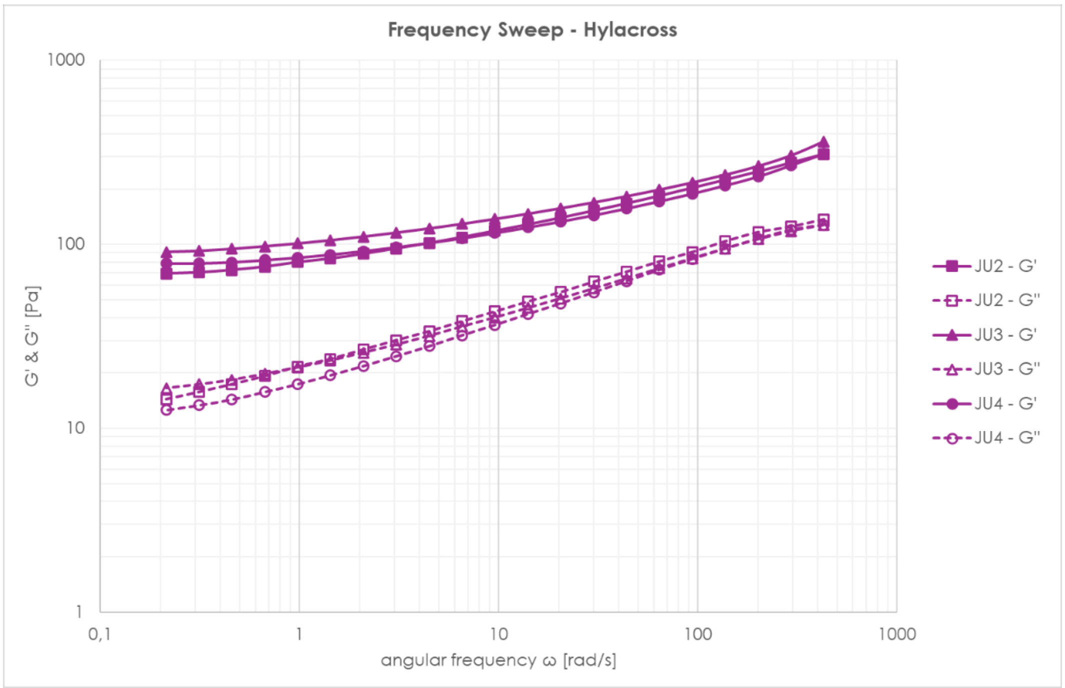


**Figure S1:** Frequency sweep of the Hylacross products.

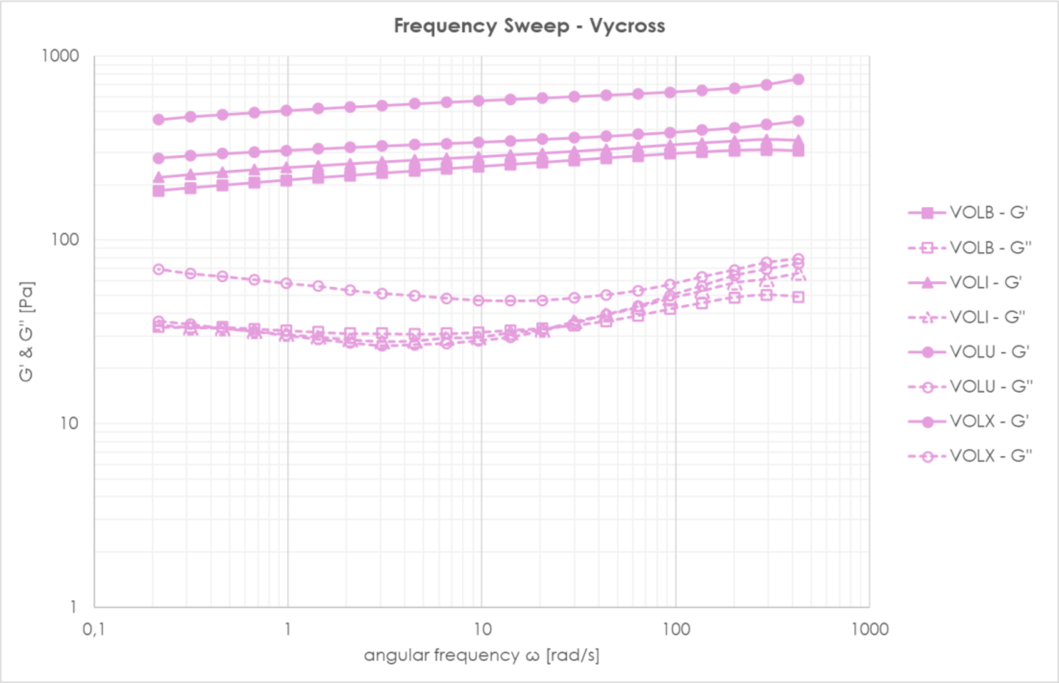


**Figure S2:** Frequency sweep of the Vycross products.

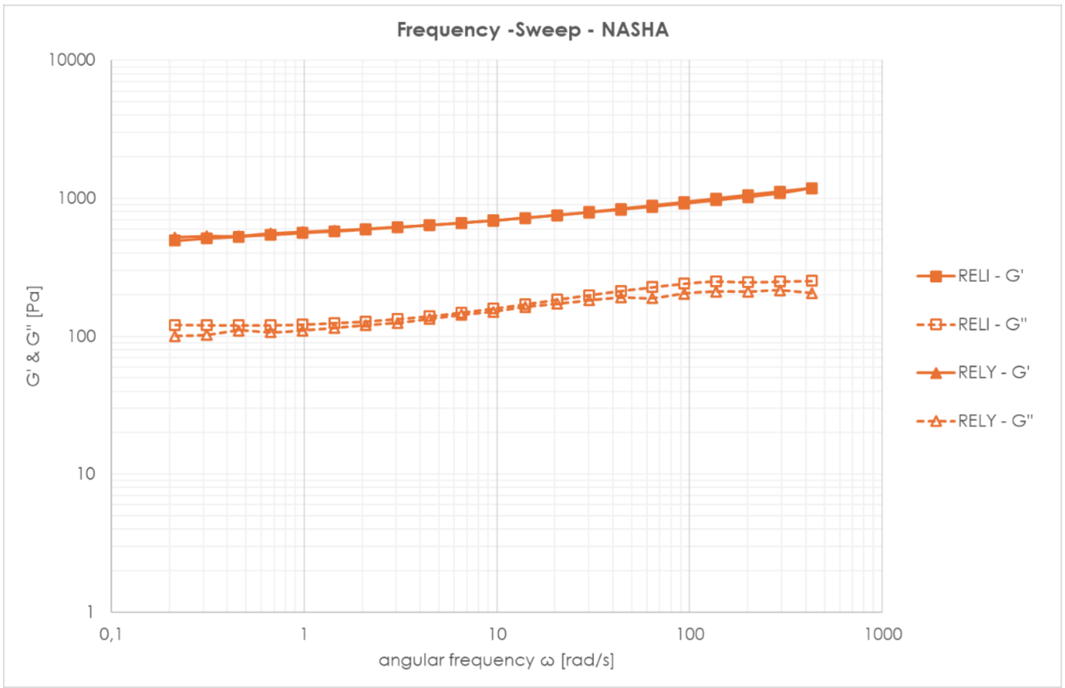


**Figure S3:** Frequency sweep of the NASHA products.

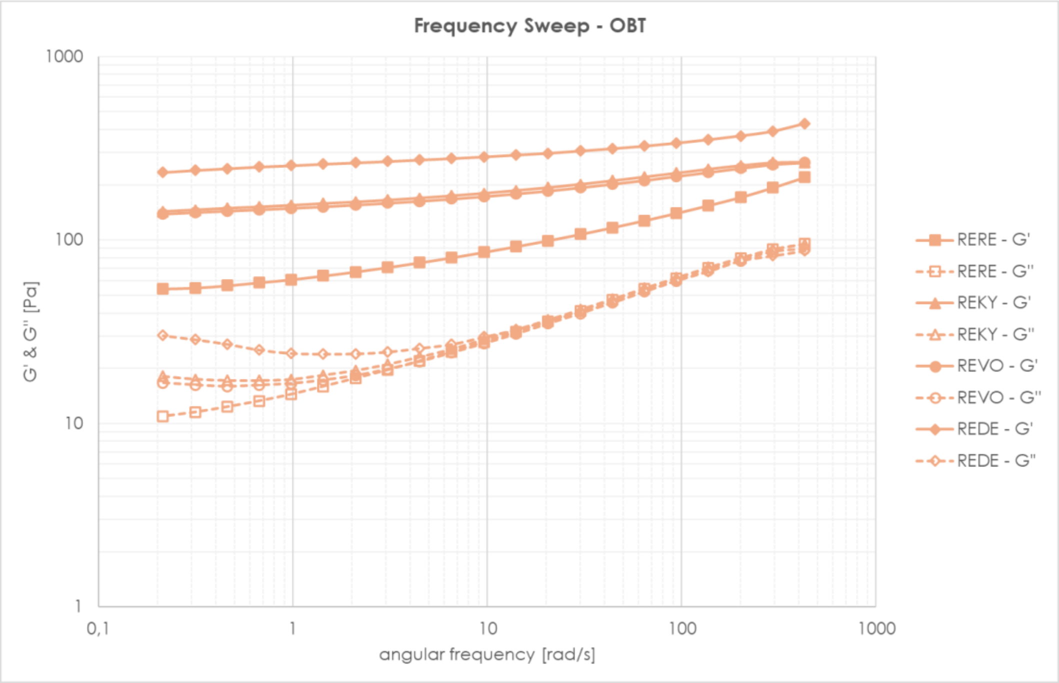


**Figure S4:** Frequency sweep of the OBT products.

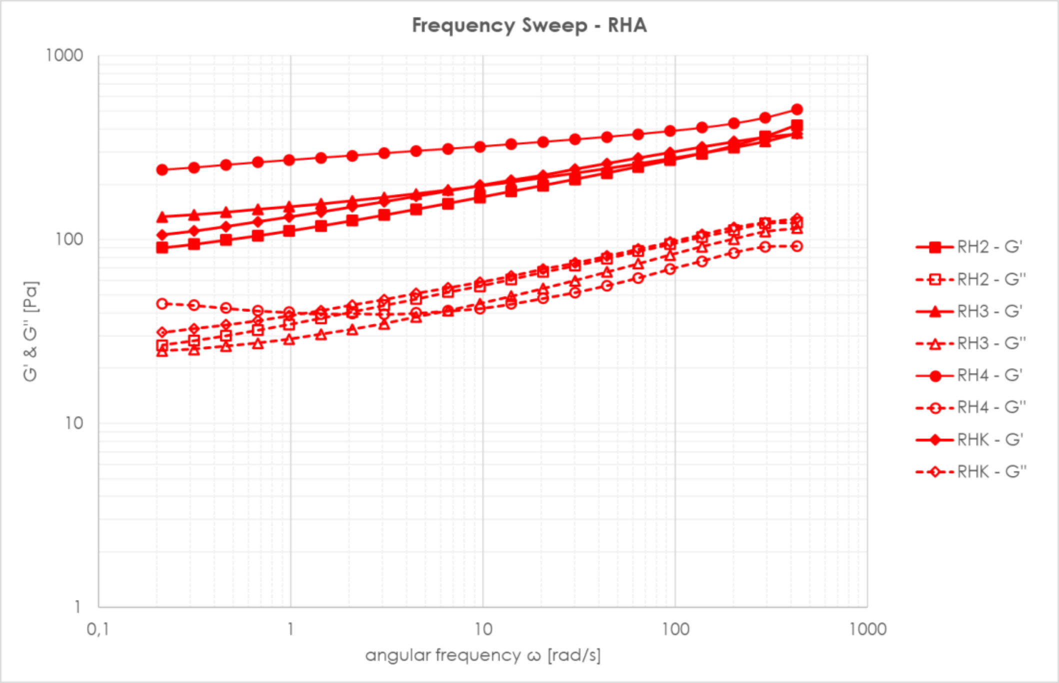


**Figure S5:** Frequency sweep of the RHA products.

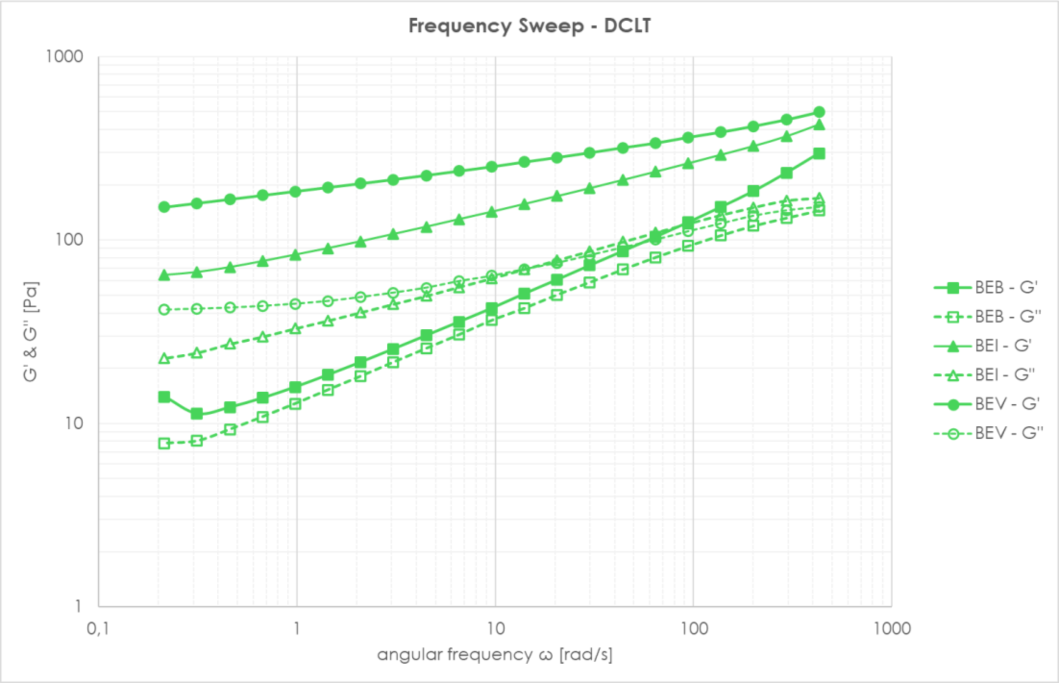


**Figure S6:** Frequency sweep of the DCLT products.

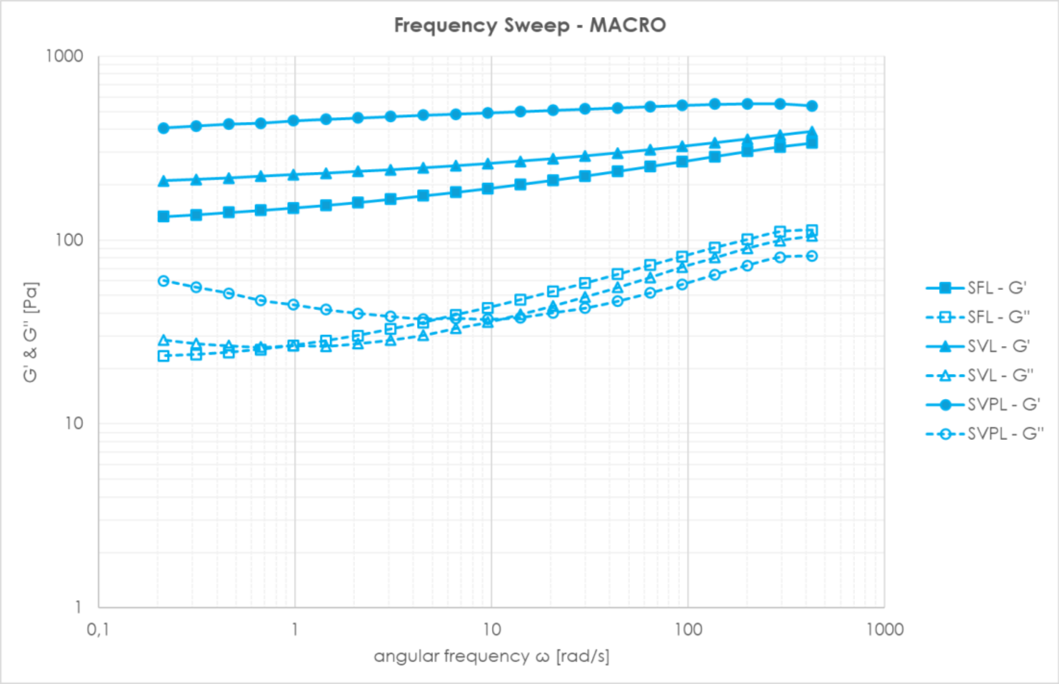


**Figure S7:** Frequency sweep of the MACRO products.

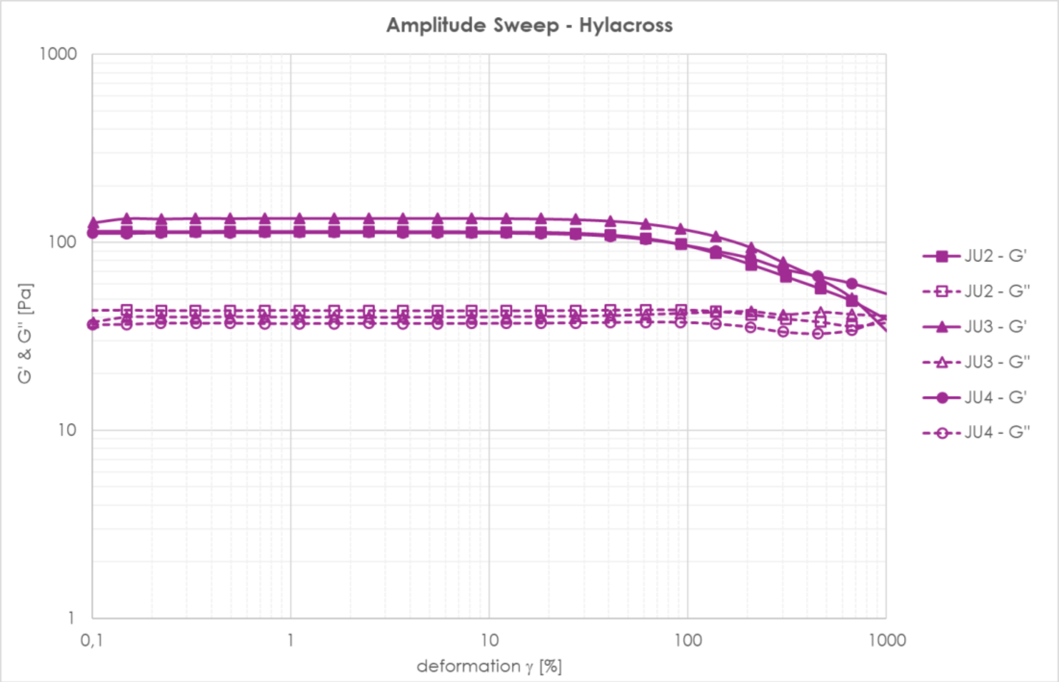


**Figure S8:** Amplitude sweep of the Hylacross products.

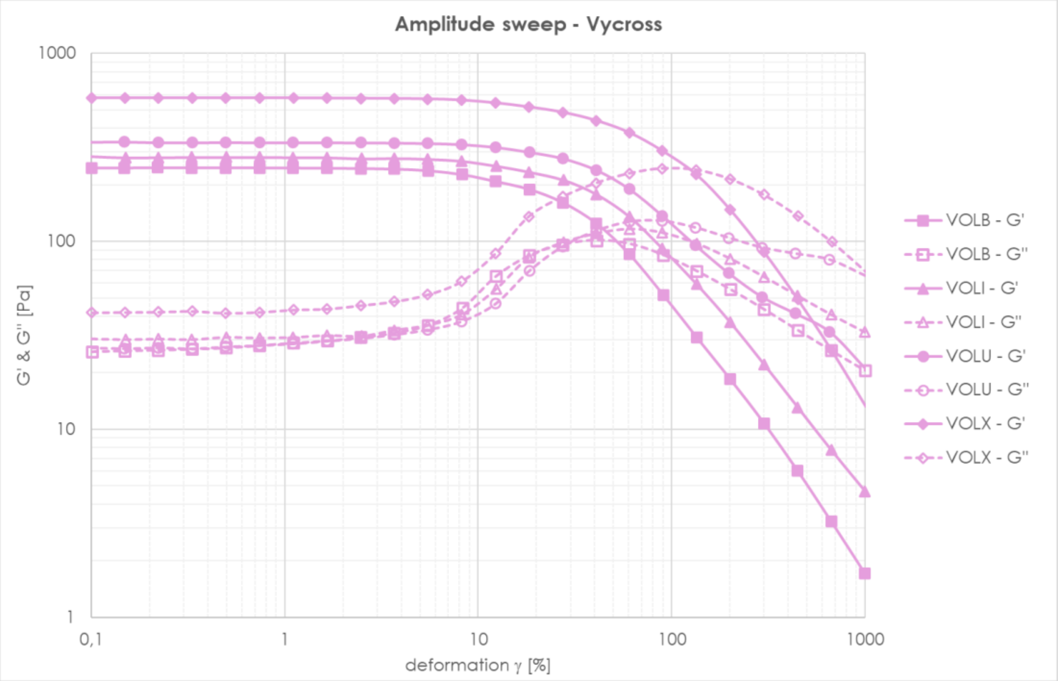


**Figure S9:** Amplitude sweep of the Vycross products.

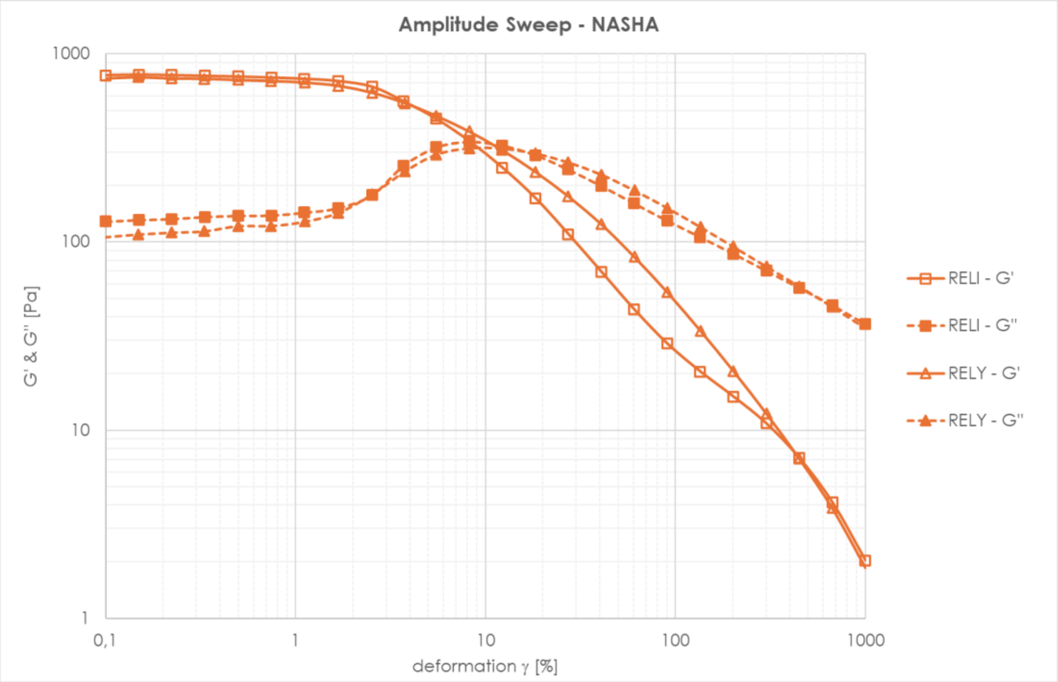


**Figure S10:** Amplitude sweep of the NASHA products.

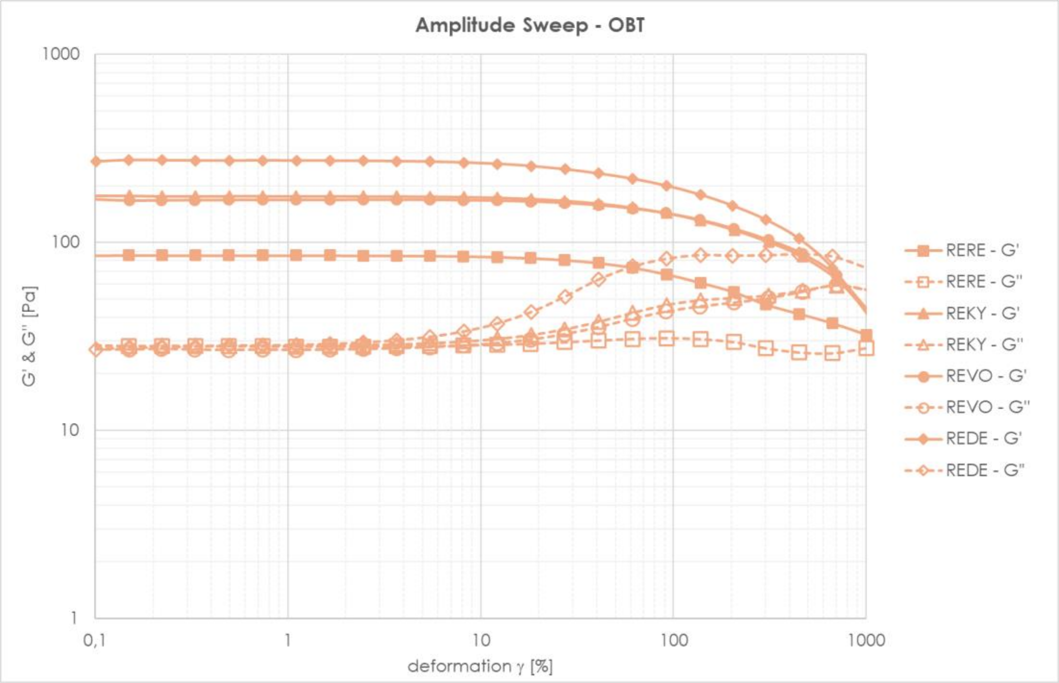


**Figure S11:** Amplitude sweep of the OBT products.

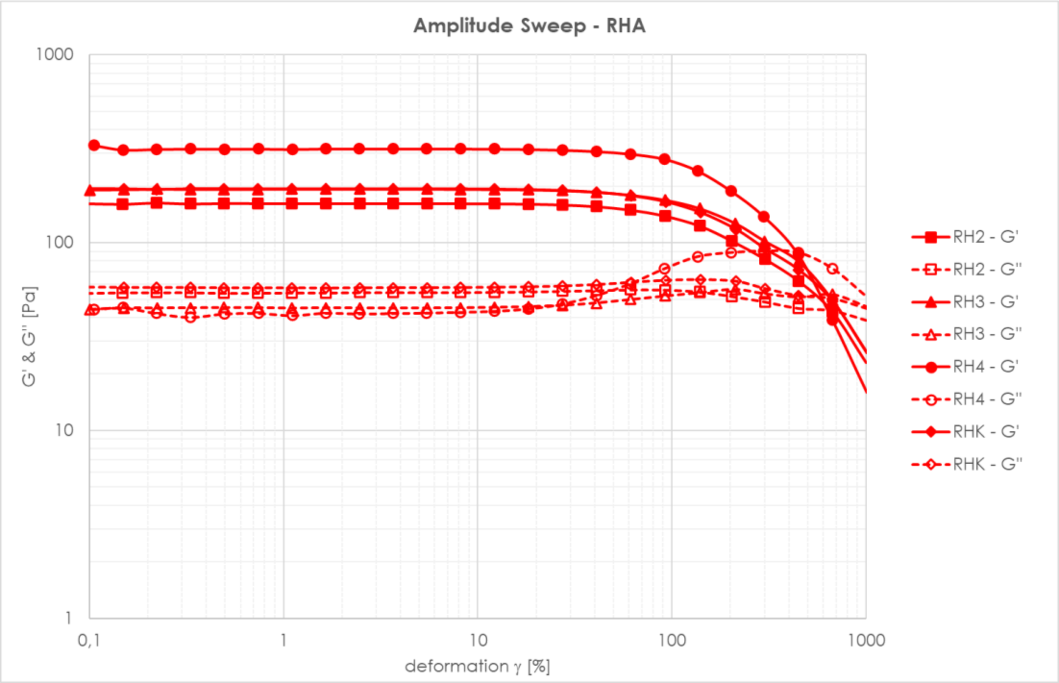


**Figure S12:** Amplitude sweep of the RHA products.

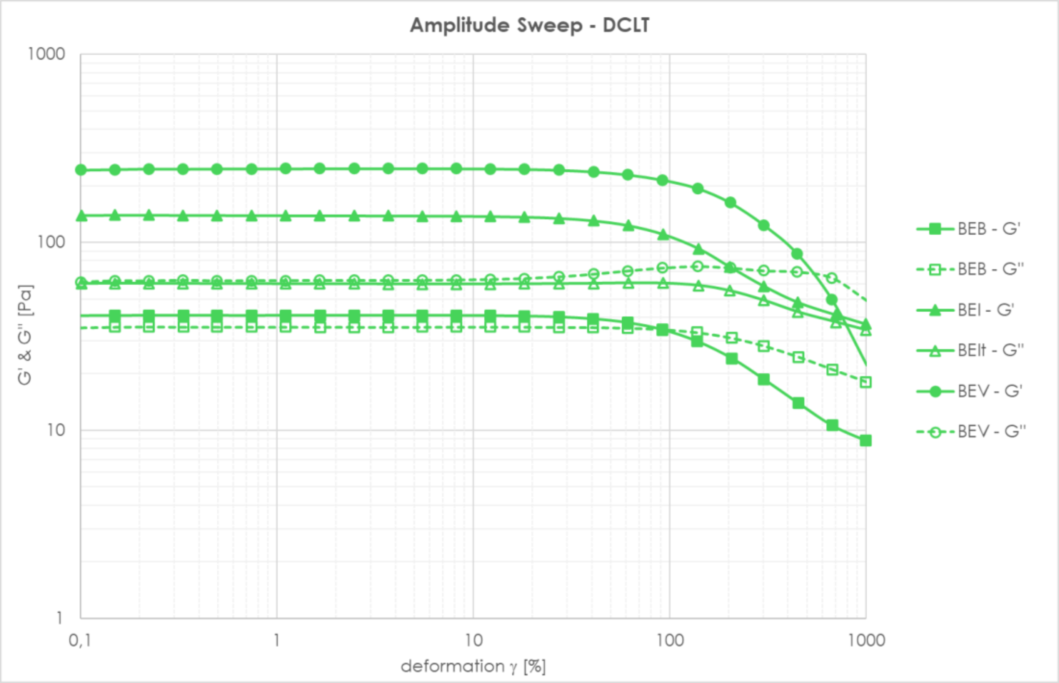


**Figure S13:** Amplitude sweep of the DCLT products.

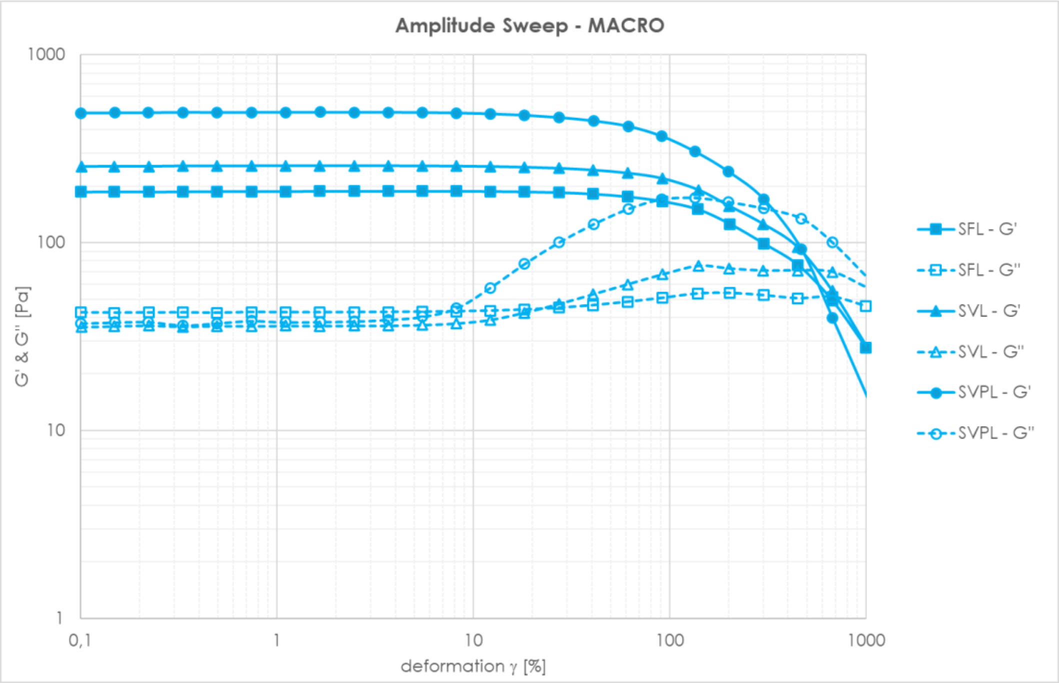


**Figure S14:** Amplitude sweep of the MACRO products.
